# Seroprevalence and Associated Factors of Small Ruminant Brucellosis in the Dubti District of the Afar Region, Ethiopia

**DOI:** 10.1155/vmi/7469192

**Published:** 2025-04-02

**Authors:** Teshager Dubie, Trhas Kasa, Getachew Kinfe, Abdallah Bulbula, Hagos Asgedom

**Affiliations:** ^1^Department of Veterinary Medicine, College of Veterinary Medicine and Animal Sciences, Samara University, P.O. Box 132, Samara, Ethiopia; ^2^Animal Health Institute (AHI), P.O. Box 19, Sebeta, Ethiopia

**Keywords:** Afar, brucellosis, ELISA, RBPT, seroprevalence, small ruminant

## Abstract

**Background:** Brucellosis is a bacterial zoonotic disease that leads to reproductive losses in animals, posing significant public health risks and economic challenges in sub-Saharan Africa, including Ethiopia. This study aims to estimate the seroprevalence and identify risk factors associated with small ruminant brucellosis in the region.

**Methods:** A cross-sectional study was conducted from February to April 2022 in six selected kebeles of the Dubti district in the Afar region of Ethiopia to investigate the seroepidemiology of ovine and caprine brucellosis. Researchers collected 300 serum samples from apparently healthy sheep (69) and goats (231). The samples were initially screened for brucella antigens using the rose bengal plate test (RBPT). Reactors identified by the RBPT were then confirmed using the indirect enzyme-linked immunosorbent assay (I-ELISA) at the Animal Health Institution (AHI). The study employed descriptive statistics, Pearson's chi-square (*X*^2^), and univariable logistic regression analyses to analyze the data.

**Results:** The overall seroprevalence of brucella infection in small ruminants in this study was 6.7% (20 out of 300). Specifically, the seroprevalence rate in sheep was 7.2% (5 out of 69), and in goats, it was 6.5% (15 out of 231). The chi-square and univariable logistic regression analyses showed no significant association between brucella seropositivity and factors such as species, sex, age, parity, district, or abortion history (*p* > 0.05).

**Conclusions:** The findings of this study reveal that brucella infection is circulating in small ruminants in the study area and the broader Afar region. Further research should be conducted across the entire region that focuses on determining the regional seroprevalence of brucella, conducting molecular-based isolation, and identifying biotypes.

## 1. Introduction

In Ethiopia, especially in pastoral and agropastoral regions, the community primarily depends on the livestock sector for essential resources such as meat, milk, draft power, and income. This reliance highlights the critical role of livestock in supporting livelihoods and driving the local economy [1, 2]. Small ruminants are desirable livestock species since they grow more quickly, require less maintenance, have shorter production cycles, and are better adaptable to harsh climates than large ruminants [3]. Despite having a huge population of small ruminants, Ethiopia is unable to make the best use of this resource due to many factors, including widespread infectious diseases, a lack of a suitable disease management strategy, inadequate feeding, poor management, a lack of genetic empowerment, and a lack of government attention [1]. Brucellosis is a significant infectious disease affecting small ruminants and is widespread across all regions of Ethiopia, hampering their productivity [4, 5]. Recognized as the world's most prevalent bacterial zoonosis, brucellosis is highly contagious and has substantial public health and economic importance. The World Health Organization (WHO) ranks it as the second-most significant zoonotic disease globally, with major economic and public health impacts, particularly in sub-Saharan Africa, including Ethiopia [6].

Brucellosis is a widespread zoonotic disease that poses serious health risks to humans and animals, especially in regions reliant on livestock for food and income [7]. In pastoral and agropastoral communities, close contact with animals and consumption of raw milk increase the risk of infection, as nearly all pastoralists drink raw milk [8, 9]. Brucella species are facultative intracellular, Gram-negative, flagellated, nonmobile, oxidase-, catalase-, and urease-positive, nonspore-forming, noncapsulated, and partially acid-fast coccobacilli, lacking endospores and native plasmids [10].

In livestock, brucellosis is primarily caused by *Brucella abortus*, *Brucella melitensis*, *Brucella suis*, *Brucella canis*, and *Brucella ovis*. In sheep and goats, *B. melitensis* (biovars 1, 2, or 3) and *B. ovis* are the main culprits [11]. These infections have significant veterinary and public health implications, leading to economic losses from abortion, neonatal death, decreased fertility, and reduced milk supply [12]. Brucellosis spreads mainly through inhalation, contact with an infected fetus, or ingestion of contaminated food and drink. Infected animals excrete large quantities of the bacteria in their urine, milk, vaginal discharge, semen, and birthing fluids [13]. Studies in Ethiopia have shown that small ruminant brucellosis is widespread, particularly in pastoral areas such as the Afar region. Contributing factors include consuming raw milk, handling contaminated animal waste, mixing the care of different animal species, and managing large herds. The prevalence of the disease in the Afar region is thought to be influenced by recurring natural disasters (such as droughts and floods), the introduction of new animals as replacement stock, and food security initiatives for vulnerable communities [14, 15]. There is a lack of up-to-date information on brucellosis in small ruminants in the current study area. Therefore, this study aims to estimate the seroprevalence of brucellosis and identify associated risk factors in the Dubti district of the Afar region, Ethiopia.

## 2. Materials and Methods

### 2.1. Description of the Study Area

The Afar region is situated in northeastern Ethiopia and is divided into five administrative zones, which are further subdivided into 35 districts and over 358 peasant associations (PAs). This study was carried out in zone one, specifically in the Dubti district and five selected kebeles within the district. The primary livelihoods in the region are pastoralism and agropastoralism.

### 2.2. Study Population

The current study was conducted on small ruminants under extensive management in five selected kebeles of Dubti district, Afar region. The study populations came from kebeles: harakis and gansuri, Ayerolef and Gebelaytu, Aredo, Debal, and Larigoh. Indigenous Afar sheep and goats were included, with all male and female small ruminants aged 6 months and older considered as study animals. Due to the absence of brucella vaccination in Ethiopia, vaccination was not taken into account. The study animals were categorized into young (6 months–2 years) and adults (over 2 years).

### 2.3. Study Design

A cross-sectional study was carried out to estimate the seroprevalence of brucellosis in small ruminants within selected kebeles of Dubti district and to assess potential risk factors associated with the disease from March to April 2022. A questionnaire focusing on variables such as age, sex, parity, abortion history, and herd size was administered to herd owners to evaluate the associated risk factors in the area.

### 2.4. Sample Size Determination

The sample size was calculated using Thrusfield's [16] method, based on an expected brucellosis prevalence of 2.4% from a previous zonal-level study by Sintayehu et al. [11]. The absolute precision and 95% confidence interval (CI) were as follows:

(1) $$\begin{array}{c} n=\frac{Z^{2}x\text{ Pexp}\left(1-P_{\exp}\right)}{d^{2}}, \end{array}$$

where *n* = required sampling units, *Z* = multiplier from normal distribution at 95% CI (1.96), *P*_exp_ = estimated (expected) prevalence 50% (0.5), (1 − *P*) = probability of having no disease 50% (0.5), and *D* = desired absolute precision 5% (0.05).

According to the formal, the total sample size was set to 36 sera, which was deemed too small for accurate representation. To improve precision and better represent the small ruminant population in the district, the sample size was increased to 300 blood sera. Sampling was proportionally distributed based on the population of small ruminants in the district's kebeles. While the number of sheep and goat samples was not proportional, the selection process was random in the field.

### 2.5. Sample Collection and Sampling Methodology

Approximately 8-9 mL of blood was drawn from the jugular vein of each sheep and goat using plain vacutainer tubes and needles, adhering to ethical guidelines outlined in the ethical declaration section. Each tube was individually labeled and kept in an icebox. The samples were left to stand overnight to allow for serum separation. The separated sera were then collected into sterile cryogenic tubes, labeled, and stored at −20°C until transported to the Animal Health Institution (AHI) in Sebeta, Ethiopia, for serological analysis. The samples were shipped in an icebox with ice packs. Relevant study data were recorded along with the blood serum collection, including details such as the identity of the animal, species, sex, age, abortion history, and parity. The zone and study district were purposively selected to get transport access as the sample for the current study was collected together with a team who came from theAHI to Dubti district for its own purpose. However, the five kebeles were randomly selected from the list of different kebeles in the district. In addition, individual animals were also randomly sampled from the herd.

### 2.6. Serological Laboratory Analysis

#### 2.6.1. Rose Bengal Plate Test (RBPT)

The modified RBPT was used as a screening method for detecting brucella agglutinins in all collected sera samples. Following the OIE [17] protocol, all sera were tested for antibodies against natural brucella infection at the bacterial serology unit of the AHI in Sebeta. The procedure involved dispensing 30 µL of stained rose bengal antigen onto a card plate, adding 75 µL of sera samples beside the antigen, and mixing them with micropipette tips. The samples were then examined for agglutination, using positive and negative controls to interpret the results. Agglutination was recorded as 0, +, ++, or +++ based on its intensity, with 0 indicating a negative result and +, ++, and +++ indicating positive results.

#### 2.6.2. Indirect Enzyme-Linked Immunosorbant Assay (I-ELISA)

All positive samples with RBPT were further subjected to the I-ELISA test as a confirmatory test at the national AHI. This kit detects anti *brucella* lipopolysaccharide antibodies in bovine sera. The I-ELISA test was performed according to the manufacture's manual. All reagents were homogenized by inversion before use, and sera samples were allowed to come to room temperature. Then, 190 μL of the dilution Buffer2 was added to all the 96 micro plate wells, and 10 μL of the negative control was added to wells A1 and B1. 10 μL of the positive control was added to wells C1 and D1, and 10 μL of sera samples to be tested was added to the remaining wells. Then, after the wells were covered with adhesive film, the plate was incubated at 37^0^c for 45 min. Then, after the content was discarded, each well was washed three times with 300 μL of the wash solution. 100 μL of the conjugate 1x was added to each well and then incubated for 30 min. Then, after the content of the wells was discarded, each well was washed three times with 300 μL of the wash solution again. 100 μL of buffered peroxide substrate was added into each well and mixed by a plate agitator to ensure correct homogenization. After incubation at room temperature for 15 min shielded from light, 100 µL of stop solution was added and mixed with the plate agitator. The sample was then put in to the ELISA reader. Finally, the result was obtained by printing from the computer connected to read under the microreader at the 450 nm ELISA reader.

### 2.7. Ethical Consideration and Consent to Participate

Ethical clearance for this study was obtained from the Animal Research Ethical and Review Committee of the AHI (ref. no: VM/ERC/10/03/12/2022). Animal owners were informed about the study's objectives and gave verbal consent for blood sample collection from their sheep and goats, which was included in the ethical clearance. During sample collection, the animals were treated according to best veterinary practices.

### 2.8. Data Management and Analysis

Data from the laboratory analysis and questionnaire survey were recorded in Microsoft Excel and analyzed using STATA version 14.0 for Windows (Stata Corp. College Station, USA). In the statistical analysis, the first level of each independent variable was used as the reference category. Seroprevalence was determined by dividing the number of sheep and goats testing positive by the I-ELISA confirmatory test by the total number of sera tested. Chi-square (X^2^) and univariable logistic regression were employed to assess the association between seropositivity and explanatory variables. A 95% CI and a significance level of 5% were used, with *p* < 0.05 considered statistically significant.

## 3. Results

In the present study, out of 300 sera collected from the study population and tested using the RBPT), 34 (11.3%) and all the positive serum samples by RBPT were tested for confirmatory by the I-ELISA. The confirmatory test revealed that 6.7% (*n* = 20/300; 95% CI of 4.15–7.95) were found to be positive for the presence of antibodies against brucella antigen infection. The demographic characteristics of the study population are presented in Table 1. The majority of the study population, 87.3% (*n* = 262), were females, while about 12.67% (*n* = 38) of them were males.

### 3.1. Seroprevalence of Brucella Infection

**Figure 1:**
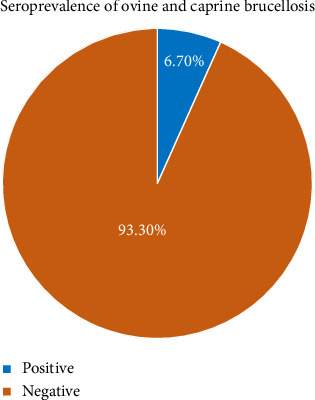
The overall seroprevalence of brucella infection in the study district of the Afar region.

Of the 300 sera samples from sheep and goats tested with the RBPT, 11.3% (*n* = 34) were positive. These positive samples were further tested with the I-ELISA, confirming 20 samples (6.7%) positive for brucella antibodies (Figure 1). The seroprevalence of brucella infection in the Afar region study district was 7.2% in sheep and 6.5% in goats. All tested sera were from female animals, with an overall prevalence of 6.7% (*n* = 20/300).

### 3.2. Associated Factors of Brucella Infection

The study assessed various risk factors for brucella infection seroprevalence, including species, sex, age, parity, district, and abortion history, using structured questionnaires for each sampled herd (Table 2). Results indicated variations in brucella infection seroprevalence across different geographical areas. Among age groups, the seroprevalence was 8.9% in younger animals and 5.9% in adults. All samples were from female animals, with a seroprevalence of 6.7%. Chi-square (*X*^2^) analysis showed no statistically significant association between any of the assessed risk factors and brucellosis seroprevalence (Table 2).

The results of the univariate logistic regression analysis indicated that species (*p* = 1.000), sex (*p* = 0.089), age group (*p* = 0.430), parity (*p* = 1.000), and age (*p* = 0.212) did not have a significant association with ELISA seropositivity, as shown in (Table 3).

## 4. Discussion

In Ethiopia, especially in pastoral areas, close human-animal interactions are common as livestock is vital for the community's livelihood. Brucellosis, a severe zoonotic bacterial pathogen, causes abortion in naturally infected small ruminants and is a significant issue globally. This disease directly and indirectly impacts human health and leads to substantial economic losses [18]. To safeguard the health of both humans and animals, it is crucial to monitor the occurrence of brucellosis in both populations. The current study found the overall seroprevalence of brucellosis in small ruminants in the study district of the Afar region to be 6.7% (*n* = 20/300), with 6.5% in goats and 7.2% in sheep. This seroprevalence aligns with previous findings: Benkirane et al. [18] and Muluken et al. [19] reported 5.6% and 7.52%, respectively, in the Afar region, Teshome et al. [20] found 2.6% in Dire Dawa, Sorsa et al. [21] estimated 5.4% in southern Ethiopia, Tsehay et al. [22] detected 5.42% in Oromia and Somali regions, Shimeles and Andualem [23] discovered 5.87% in Amhara, Dabasa et al. [24] reported 1.17% in sheep and 1.88% in goats in Borena, Sintayehu et al. [11] found 3.3% in small ruminants, and Nigatu et al. [25] reported 6.7% in selected export abattoirs in Ethiopia. The findings of this study are consistent with those of other countries, such as Nigeria, where Bertu et al. [26] reported a 5.2% seroprevalence. However, the seroprevalence observed in the current study is higher than that reported in various regions of Ethiopia. For example, Sintayehu et al. [11] recorded a prevalence of 2.4% in Zone 1 of the Afar region; Yesuf et al. [27] and Ferede et al. [4] found 0.4% and 0.2% in Bahir Dar; Girmay et al. [28] reported 0.9% in Somali and Oromia and 0.53% in Bale and Boran; found 1.7% in goats and 1.6% in sheep in the Somali region; Tsehay et al. [22] reported 1.74% of small ruminants brucellosis seroprevalence in Oromina and Somalia regions; Aregawi, Daniel, and Berihun [29] estimated 0.6%; and Mihreteab et al. [30] reported 1.56% in Jijiga District, Somali Regional State; Tewodros and Dawit [31] also reported 0.7% in Kombolcha, Northeastern Ethiopia; Umer et al. [32] recorded 0.24% of brucella seroprevalence in the West Hararghe zone, Oromia region; Yeshibelay and Teferi [33] also reported 0.78% of small ruminant brucellosis seroprevalence in Eastern Hararghe of the Oromia region, Ethiopia. In contrast to the findings of the current study, previous research has reported higher seroprevalence rates of small ruminant brucellosis. For example, Tadeg et al. [34] found a seroprevalence of 13.6% in the Tallalak area of Afar, and Tegegn et al. [35] recorded 13.7% in Chifra and Ewa districts of Afar. Other studies reported 12.4% by Adugna et al. [36]; 16% by Anteneh [37]; and 9.11% by Negash et al. [38] in areas under Dire Dawa administration. Differences in seroprevalence rates between these studies and the current one can be attributed to several factors: variations in agroecological conditions, the composition of the sampled animals, the sensitivity and specificity of the serological tests used, management practices, sample sizes, breeds of small ruminants, flock sizes, reintroduction of animals from brucellosis-prevalent areas, and sampling methods. Although goats were reported to have a higher prevalence of brucellosis than sheep, the difference was not statistically significant (*p* > 0.05). However, Adugna et al. [36] and Teshale et al. [39] found the seroprevalence to be significantly higher (*p* < 0.05) in goats than in sheep. This higher prevalence in goats may be due to their greater susceptibility to brucella infection and the fact that sheep do not excrete brucella organisms for as long, reducing the disease's potential spread among sheep flocks.

The current study found that only female sheep and goats had positive serum results using the I-ELISA. This could be attributed to the fact that fewer males (*n* = 38) were tested compared to females (*n* = 262), and males are generally more resistant to brucella infection [40]. Hirsh and Zee [41] noted that the absence of erythritol in males makes them less susceptible to the infection. Additionally, Crawford et al. [42] observed that male animals have limited serological responses to brucella, resulting in testes that are typically nonreactors or have low antibody titers.

The seroprevalence of brucellosis was higher in adult animals compared to younger ones, although this difference was not statistically significant. This may be due to increased susceptibility following sexual maturity, particularly after pregnancy, as the presence of erythritol and other substances in the uterus, placenta, and fetal fluids promotes the growth of *B. melitensis*, the main organism causing infection in sheep and goats [43]. This finding aligns with previous studies by Ashenfi et al. [44], who reported a prevalence of 5.3% in adult animals and 1.5% in younger sheep and goats.

It has been documented that sexually immature animals of both sexes are more likely to become infected with brucella than their more sexually mature counterparts. The concentration of erythritol and sex hormones, which increases with age and sexual maturity, encourages the growth and multiplication of brucella organisms (Radositits, 2000). The current study found no statistically significant association (*p* > 0.05) between parties and the seroprevalence of the disease. However, Yohannes et al. [45] and Anteneh [37] suggested that seropositivity in female sheep and goats with no parity history may be due to repeated exposure to parturition and other physiological stressors, which increases the likelihood of brucella infection.

In this study, brucella seropositivity was more frequently observed among sheep and goats with a history of abortion (6.3%) compared to those without (1.5%). However, the difference was not statistically significant (*p* > 0.05), suggesting that brucellosis may not be associated with abortion in these animals in the study areas. Abortion in sheep and goats can have multiple infectious causes other than brucellosis, and brucella infection seropositivity is not always linked to abortion. Confirming brucella as the cause of abortion requires identifying the pathogen in the tissues of aborted fetuses or placentas, as well as in the aborting animals [46].

This finding aligns with studies by Tulu et al. [47], Gebremedhin [48], and Wubish et al. [49], who found no association between brucella seropositivity and abortion. However, it contradicts other Ethiopian studies [50, 51] that reported an association. These variations could be due to differences in regional agroecology, breed, management, husbandry practices, and factors that facilitate the spread of various causes of abortion [52].

## 5. Conclusions

The present study revealed that the seroprevalence of small ruminant brucellosis in the study area was relatively low compared to other findings in the Afar region but still higher than that recorded in other regions of Ethiopia. Therefore, it is crucial to conduct further studies to identify the circulating brucella species using molecular tools and investigate other potential causes of abortion in sheep and goats. Additionally, this study highlights the need for further research on brucella infection, its public health impact, and its effects on reproduction and production in the area. Future studies should cover more districts with larger sample sizes to better understand the prevalence of small ruminant brucellosis in the region and the country as a whole.

## Figures and Tables

**Table 1:** The demographic characteristics of the study animals.

Variables	Category	Sample size	Percentage (%)
Sex	Male	38	12.67
	Female	262	87.3

Species	Ovine	69	23
	Caprine	231	77

Age	Young	79	26.3
	Adult	221	73.66

Parity	Yes	115	38.33
	No	185	61.66

Abortion history	Yes	13	4.33
	No	287	95.66

District	1. Harakis and Gansuri	60	2 (3.3)
	2. Ayerolef and Gebelaytu	60	3 (5)
	3. Aredo and Larigoh	60	6 (10)
	4. Debal	44	1 (2.3)
	5. 01	76	8 (10.5)

**Table 2:** Chi-square analysis results of the associated factor of brucellosis seroprevalence.

Variables	Category	Sample size	Seropositive%	Chi-square (*X*^2^)	*p* value
Sex	Male	38	0 (0)	3.1080	0.078
	Female	262	20 (7.6)		

Species	Ovine	69	5 (7.2)	0.484	0.826
	Caprine	231	15 (6.5)		

Age	Young	79	7 (8.9)	0.8297	0.362
	Adult	221	13 (5.9)		

Parity	Yes	115	12 (6.5)	0.0252	0.874
	No	185	8 (6.9)		

Abortion history	Yes	287	18 (6.3)	1.6598	0.198
	No	13	2 (1.5)		

**Table 3:** Univariable analysis results of associated factors for brucellosis seroprevalence.

Variables	Category	Seropositive	Seroprevalence (%)	*p* value	95% CI
Sex	Male	0	0	0.089	0.000–1.765
	Female	20	100	Ref	Ref

Species	Ovine	5	25	1.000	0.416–3.909
	Caprine	15	75	Ref	Ref

Age	Young	7	35	0.430	0.228–3.057
	Adult	13	65	Ref	Ref

Parity	Yes	8	40	1.000	0.185–2.291
	No	12	60	Ref	Ref

Abortion history	Yes	18	90	Ref	Ref
	No	2	10	0.212	0.409–10.347

## Data Availability

The datasets generated and/or analyzed during the current study are not publicly available due to the confidentiality agreements made by all authors but could be available from the corresponding author on reasonable request.
